# Cancer-derived cholesterol sulfate is a key mediator to prevent tumor infiltration by effector T cells

**DOI:** 10.1093/intimm/dxac002

**Published:** 2022-01-30

**Authors:** Takaaki Tatsuguchi, Takehito Uruno, Yuki Sugiura, Daiji Sakata, Yoshihiro Izumi, Tetsuya Sakurai, Yuko Hattori, Eiji Oki, Naoto Kubota, Koshiro Nishimoto, Masafumi Oyama, Kazufumi Kunimura, Takuto Ohki, Takeshi Bamba, Hideaki Tahara, Michiie Sakamoto, Masafumi Nakamura, Makoto Suematsu, Yoshinori Fukui

**Affiliations:** Division of Immunogenetics, Department of Immunobiology and Neuroscience, Medical Institute of Bioregulation, Kyushu University, Fukuoka, Japan; Department of Surgery and Oncology, Graduate School of Medical Sciences, Kyushu University, Fukuoka, Japan; Division of Immunogenetics, Department of Immunobiology and Neuroscience, Medical Institute of Bioregulation, Kyushu University, Fukuoka, Japan; Department of Biochemistry, Keio University School of Medicine, Tokyo, Japan; Division of Immunogenetics, Department of Immunobiology and Neuroscience, Medical Institute of Bioregulation, Kyushu University, Fukuoka, Japan; Division of Metabolomics, Research Center for Transomics Medicine, Medical Institute of Bioregulation, Kyushu University, Fukuoka, Japan; Division of Immunogenetics, Department of Immunobiology and Neuroscience, Medical Institute of Bioregulation, Kyushu University, Fukuoka, Japan; Department of Biochemistry, Keio University School of Medicine, Tokyo, Japan; Department of Surgery and Science, Graduate School of Medical Sciences, Kyushu University, Fukuoka, Japan; Department of Pathology, Keio University School of Medicine, Tokyo, Japan; Department of Uro-Oncology, Saitama Medical University International Medical Center, Saitama, Japan; Department of Uro-Oncology, Saitama Medical University International Medical Center, Saitama, Japan; Division of Immunogenetics, Department of Immunobiology and Neuroscience, Medical Institute of Bioregulation, Kyushu University, Fukuoka, Japan; Department of Biochemistry, Keio University School of Medicine, Tokyo, Japan; Division of Metabolomics, Research Center for Transomics Medicine, Medical Institute of Bioregulation, Kyushu University, Fukuoka, Japan; Department of Cancer Drug Discovery and Development, Research Center, Osaka International Cancer Institute, Osaka, Japan; Project Division of Cancer Biomolecular Therapy, Institute of Medical Science, The University of Tokyo, Tokyo, Japan; Department of Pathology, Keio University School of Medicine, Tokyo, Japan; Department of Surgery and Oncology, Graduate School of Medical Sciences, Kyushu University, Fukuoka, Japan; Department of Biochemistry, Keio University School of Medicine, Tokyo, Japan; Division of Immunogenetics, Department of Immunobiology and Neuroscience, Medical Institute of Bioregulation, Kyushu University, Fukuoka, Japan

**Keywords:** DOCK2, immune evasion, SULT2B1b, tumor immunotherapy

## Abstract

Effective tumor immunotherapy requires physical contact of T cells with cancer cells. However, tumors often constitute a specialized microenvironment that excludes T cells from the vicinity of cancer cells, and its underlying mechanisms are still poorly understood. DOCK2 is a Rac activator critical for migration and activation of lymphocytes. We herein show that cancer-derived cholesterol sulfate (CS), a lipid product of the sulfotransferase SULT2B1b, acts as a DOCK2 inhibitor and prevents tumor infiltration by effector T cells. Using clinical samples, we found that CS was abundantly produced in certain types of human cancers such as colon cancers. Functionally, CS-producing cancer cells exhibited resistance to cancer-specific T-cell transfer and immune checkpoint blockade. Although SULT2B1b is known to sulfate oxysterols and inactivate their tumor-promoting activity, the expression levels of cholesterol hydroxylases, which mediate oxysterol production, are low in SULT2B1b-expressing cancers. Therefore, SULT2B1b inhibition could be a therapeutic strategy to disrupt tumor immune evasion in oxysterol-non-producing cancers. Thus, our findings define a previously unknown mechanism for tumor immune evasion and provide a novel insight into the development of effective immunotherapies.

## Teaser

The roles of sulfation in immune responses to tumors

## Graphical abstract

**Graphical abstract:**
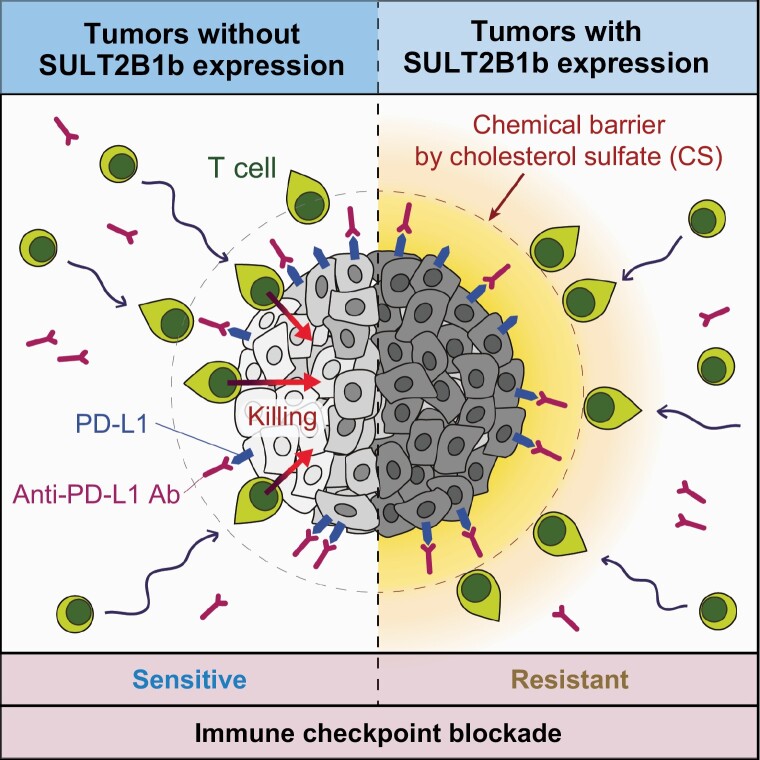

## Introduction

Although immunotherapies, such as immune checkpoint blockade, have revolutionized cancer treatment, many patients still do not derive clinical benefits (1). This resistance to immunotherapy could be caused by several tumor-intrinsic mechanisms that affect the generation and function of cancer-specific T cells. They include a lack of antigenic mutations, disruption of the antigen-processing machinery, loss of major histocompatibility complex (MHC) expression, and defects in interferon-γ (IFN-γ)- or tumor necrosis factor (TNF)-mediated signaling pathway that acts to increase tumor immune susceptibility (2–5). On the other hand, tumors often constitute specialized microenvironments that exclude T cells from the vicinity of cancer cells, even though functional cancer-specific T cells exist in the draining lymph nodes (6, 7). As effective tumor immunotherapy requires physical contact of T cells with cancer cells, elucidation of the mechanisms is required for the development of better therapies. So far, it has been proposed that epigenetic silencing of the genes encoding chemokines in the tumor microenvironments and oncogenic pathways in tumors such as WNT/β-catenin signaling are associated with T-cell exclusion (4, 8–11). However, other mechanisms underlying the prevention of tumor infiltration by T cells are largely unknown.

DOCK2 is a mammalian homolog of *Caenorhabditis elegans* CED-5 and *Drosophila melanogaster* Myoblast City, and is predominantly expressed in hematopoietic cells (12, 13). Although DOCK2 does not contain the Dbl homology (DH) domain that is typically found in guanine nucleotide exchange factors (GEFs), DOCK2 mediates the GTP–GDP exchange reaction for Rac via its DOCK homology region (DHR)-2 domain (14, 15). Accumulating evidence in mice indicates that DOCK2 is a major Rac GEF acting downstream of chemoattractant receptors and antigen receptors in lymphocytes and plays key roles in their migration and activation (13, 16–19). In addition, it has been shown that *DOCK2* mutations cause severe immunodeficiency in humans (20). Thus, DOCK2 is essential for immune surveillance mechanisms in humans and mice.

Sulfation plays an important role in the regulation of the biological activity of many endogenous molecules. We earlier reported that cholesterol sulfate (CS), but not cholesterol, acts as an endogenous inhibitor of DOCK2 (21). Indeed, CS directly binds to the catalytic DHR-2 domain of DOCK2, and inhibits its Rac GEF activity (21). This inhibitory effect is specific for CS, and no such effect was seen for other cholesterol derivatives (21). In both humans and mice, sulfation of cholesterol is mediated by the sulfotransferases SULT2B1b and, to a lesser extent, SULT2B1a, which are produced from the same gene, *SULT2B1*, through alternative splicing (21–24). However, SULT2B1b is also known to sulfate and inactivate oxysterols, cholesterol-oxidized products that have been shown to favor tumor growth by directly promoting tumor cell growth and indirectly by dampening anti-tumor immune responses (25–27). Therefore, the expression of SULT2B1b in tumors would affect anti-tumor responses differently in oxysterol-producing tumors and oxysterol-non-producing tumors.

In this study, we show that CS is abundantly produced in certain types of human cancer, such as colon cancer, and CS-producing cancer cells exhibit resistance to cancer-specific T-cell transfer and immune checkpoint blockade unless they produce oxysterols. Thus, our findings define a previously unknown mechanism of tumor immune evasion and provide a novel insight into the development of effective immunotherapies.

## Methods

### Human tissue samples

Human cancer tissue samples and normal colon tissue samples were obtained during surgery from patients in compliance with Institutional Review Board protocols of Kyushu University Hospital, Keio University Hospital or Saitama Medical University Hospital. Immediately after resection during surgery, the specimens were placed in liquid nitrogen and were stored for analyses. The study protocol was approved by the institutional review board at each participating institution. Informed consent was obtained by an opt-out/opt-in approach, according to each participating institution’s policy.

### Cancer cell lines and reagents

E0771, Pan02 and E.G7-OVA cells were purchased from CH3 BioSystems (Amherst, NY, USA), National Cancer Institute and ATCC (Manassas, VA, USA), respectively. 3LL cells were obtained from Japanese Collection of Research Bioresources (Osaka, Japan), and MC38 cells were a gift from Dr S. A. Rosenberg (National Cancer Institute, Bethesda, MD, USA) and were previously reported elsewhere (28). E0771, Pan02, E.G7-OVA and 3LL cells were maintained in RPMI-1640 medium (FUJIFILM Wako Pure Chemical Corporation, Osaka, Japan) and MC38 cells were maintained in Dulbecco’s modified Eagle medium (DMEM) (FUJIFILM Wako Pure Chemical Corporation), both of which contained 10% heat-inactivated fetal bovine serum (FBS; Life Technologies, Breda, the Netherlands), 50 µM 2-mercaptoethanol (Nacalai Tesque, Kyoto, Japan), 2 mM l-glutamine (Life Technologies), 100 U ml^−1^ penicillin (Life Technologies), 100 µg ml^−1^ streptomycin (Life Technologies), 1 mM sodium pyruvate (Life Technologies) and 1× MEM non-essential amino acids (Life Technologies) (complete RPMI or DMEM medium). The culture supernatant of MC38 and its derivatives was obtained by seeding 5 × 10^5^ cells in 24-well plates and harvesting the supernatants after 30 h when the cells were confluent. 25-hydroxycholesterol (25-HC) and 25-hydroxycholesterol-3-sulfate (25-HCS) were purchased from Focus Biomolecules (Plymouth Meeting, PA, USA) and Avanti Polar Lipids (Alabaster, AL, USA), respectively.

### Mice and treatments

C57BL/6 mice and BALB/c nude mice were purchased from Japan Clea (Tokyo, Japan). *Sult2b1*^*–/–*^ mice were obtained from the Jackson Laboratory (stock no. 018773, Bar Harbor, ME, USA), which had been backcrossed with C57BL/6 mice more than seven generations before use (21). DOCK2^–/–^ mice on a C57BL/6 genetic background have been described previously (13) and were used in some experiments after crossing with OTII T-cell receptor (TCR) Tg mice. Mice were maintained under specific pathogen-free conditions in the animal facility of Kyushu University. The animal experiment protocol was approved by the committee of Ethics of Animal Experiments, Kyushu University. When indicated, 200 µl of anti-mouse PD-L1 (programmed cell death ligand 1) antibody (10F.9G2, 1 mg ml^−1^, BioXCell, West Lebanon, NH, USA) or isotype-matched control (LTF-2, 1 mg ml^−1^, BioXCell) was injected intra-peritoneally into tumor-bearing mice. For T-cell transfer, activated OTII CD4^+^ T (2 × 10^6^) and OTI CD8^+^ T cells (5 × 10^5^) were injected intravenously into tumor-bearing mice.

### Transfectants

E0771-SULT and 3LL-SULT cells were prepared by retrovirus infection of the pMX vectors encoding mouse *Sult2B1b* with an HA-tag at its C-terminus (pMX-SULT2B1b-IRES-GFP and pMX-SULT2B1b-puromycin, respectively). E0771-MOCK cells were prepared by retrovirally infecting E0771 cells with pMX-IRES-GFP. The plasmids were transfected into Platinum-E packaging cells using FuGENE 6 transfection reagent (Promega, Madison, WI, USA). The cell culture supernatants were harvested 48 h after transfection, supplemented with 5 µg ml^−1^ polybrene and used to infect E0771 and 3LL cells. For the development of E.G7-OVA-SULT and MC38-SULT, the pSI-puromycin vector encoding mouse *Sult2B1b* (pSI-SULT2B1b-puromycin) was transfected into E.G7-OVA and MC38 cells using Amaxa Nucleofector™ II (for E.G7-OVA) or polyethylenimine (for MC38). E.G7-OVA-MOCK has been developed by transfection of the pSI-puromycin vector into parental cells. Then, clones were isolated by limiting dilution, following selection with (4 µg ml^−1^ for 3LL and E.G7-OVA; 2 µg ml^−1^ for MC38) or without (E0771) puromycin.

For the expression of PD-L1, the pBJ-*neo* vector encoding mouse PD-L1 was used. To express the I-A^b^/OVA complex, cells were transfected with the pBJ-*neo* vector encoding mouse I-A^b^ α along with the pCAG vector encoding I-A^b^ β covalently bound to OVA peptide (OVA323–339: ISQAVHAAHAEINEAGR) at a 1:5 ratio using polyethylenimine. After selection with G418 (1 mg ml^−1^), clones with the comparable expression of PD-L1 or the I-A^b^/OVA complex were selected and used. For expression of OVA protein, the pBJ-*neo* vector encoding OVA with a FLAG tag was transfected into E0771-control or E0771-SULT cells. Clones were isolated by limiting dilution, following selection with 1 mg ml^−1^ of G418.

### Knockout of cholesterol 25-hydroxylase or Sult2b1

To genetically inactivate the expression of cholesterol 25-hydroxylase (CH25H) or SULT2B1b in MC38 or Pan02 cells, respectively, the CRISPR-Cas9 genome editing system was used in combination with gene targeting by homologous recombination (HR).

For the *Ch25h* gene, a target guide RNA sequence was selected within the single exon using the CHOPCHOP web tool (https://chopchop.rc.fas.harvard.edu/). Two complementary oligonucleotides (5′-CACC GATCTTGTAGCGTCGCAGGA-3′ and 5′-AAACTCCTGCGACGCTACAAGATC-3′) containing the target sequence (underlined) and *Bbs* I ligation adapters were synthesized, annealed and ligated into the *Bbs* I-digested pX330 vector to generate the single guide RNA (sgRNA) vector. To construct the HR targeting vector, the 5′ and 3′ homology arms (961 and 1084 bp) were amplified from genomic DNA isolated from MC38 cells by PCR with the following primers:

Target 5′-arm_F: 5′- ACCGGTACCTGTGAGGTTAGAGCCCAGTTCCAG-3′,
Target 5′-arm_R: 5′- ACCGCGGCCGCACCCGGACTTCTCG CCTTC -3′,
Target 3′-arm_F: 5′- TCCGGATCCTGGGGACCCAGGGATACA GGAC -3′,
Target 3′-arm_R: 5′- GACGTCGACCAGTGTCTGAAAGGGTATCCAGG -3′.

The amplified DNAs were inserted into the *Kpn* I-*Not* I and *BamH* I- *Sal* I sites of the HR510PA-1 targeting vector (System Biosciences, Mountain View, CA, USA), which carries the RFP expression cassette and hygromycin resistance gene between the homology arms. MC38 cells were transfected with 1.25 µg of sgRNA vector and 1.25 µg of HR targeting vector using 10 µl of Lipofectamine 2000 (Invitrogen, Carlsbad, CA, USA) in 60 mm culture dishes. Forty-eight hours after transfection, cells were selected with hygromycin (200 µg ml^−1^) and cloned by limiting dilution. Cells in which the expression of *Ch25h* was genetically inactivated (designated MC38-ΔCH25H cells) were identified by reverse transcription quantitative PCR (RT-qPCR), and the lack of 25-HC production was confirmed by mass spectrometry.

For the *Sult2b1* gene, a target guide RNA sequence was selected within the fourth exon of the gene using the CHOPCHOP web tool. Two complementary oligonucleotides (5′-CACCGTGCATACAGGTGATTTACGT-3′ and 5′-AAACACGTAAATCACCTGTATGCAC-3′) containing the target sequence (underlined) and *Bbs* I ligation adapters were synthesized, annealed and ligated into the *Bbs* I-digested pX330 vector to generate the sgRNA vector. To construct the HR targeting vector, the 5′ and 3′ homology arms (1684 and 1917 bp) were amplified from genomic DNA isolated from Pan02 cells by PCR with the following primers:

Target 5′-arm_F: 5′- CACTTCTTCCAACAAGAAGTAGAATTCTAATCC -3′,
Target 5′-arm_R: 5′- TCTAGATCTGACAGAGGGGAATGGAGAAGGTG -3′,
Target 3′-arm_F: 5′- CAAGGGCTGGATCCGGATGCAGAACC -3′,
Target 3′-arm_R: 5′- GACGTCGACCAACATTCCCACCCTGGTCCACCAGAC -3′.

The amplified DNAs were inserted into the *EcoR* I-*Bgl* II and *BamH* I- *Sal* I sites of the HR410PA-1 targeting vector (System Biosciences), which carries the GFP expression cassette and puromycin resistance gene between the homology arms. Pan02 cells were transfected with 1.25 µg of sgRNA vector and 1.25 µg of HR targeting vector using 10 µl of Lipofectamine 2000 (Invitrogen) in 60 mm culture dishes. Forty-eight hours after transfection, cells were selected with puromycin (2 µg ml^−1^) and cloned by limiting dilution. Cells with genetically inactivated SULT2B1b expression were identified by western blotting.

### Western blotting

Total cell lysates (30 µg) were separated by sodium dodecyl sulfate–polyacrylamide gel electrophoresis (SDS–PAGE), and immunoblotted with rabbit anti-SULT2B1b antibody (1:1000 dilution) (21) or goat anti-actin antibody (I-19, 1:1000 dilution; Santa Cruz, CA, USA), followed by incubation with horseradish peroxidase (HRP)-conjugated secondary antibodies (1:2000 dilution; Santa Cruz).

### RT-qPCR

Total RNA was isolated using ISOGEN (Nippon Gene, Tokyo, Japan). After treatment with RNase-free DNase I (Thermo Fisher Scientific), RNA samples (~1 µg) were reverse-transcribed with oligo(dT) primers (Thermo Fisher Scientific) and SuperScript III reverse transcriptase (Thermo Fisher Scientific) for amplification by PCR. Quantitative PCR was performed using a CFX Connect Real-Time PCR Detection System (BioRad, Hercules, CA, USA) with the SYBR Green PCR Master Mix (Thermo Fisher Scientific) according to the manufacturer’s instructions. The primers for *Ch25h* were supplied by Prime PCR™ PreAmp for SYBR® Green Assays from BioRad. For *Gapdh*, 5′-TGTGTCCGTCGTGGATCTGA-3′ and 5′-TTGCTGTTGAAGTCGCAGGAG-3′ were used. The expression of target genes was normalized to that of *Gapdh*. Comparison of *SULT2B1b* expression among human colon cancer tissues and mouse cell lines was performed by quantitative PCR using the plasmid DNA encoding human or murine SULT2B1b as a standard (21). Briefly, a series of a 1:4 dilution of the plasmid DNA was amplified to obtain a linear standard curve for Cq (threshold cycle) to M[Log (-2)] (*R*^2^ > 0.97) using the primer pairs 5′-ATGACATCTCGGAAATCAGCCA-3′ and 5′-GCACATCTTGGGTGTTCTCCG-3′ for human *SULT2B1b*, and 5′-CCCTGTGGAGCTCGTCTGAG-3′ and 5′-GACTGAGGCTCTCCGGTGAG-3′ for mouse *Sult2B1b*. The concentration of target genes in each sample was determined using the standard curve, and normalized by dividing with the amount of total cDNA synthesized.

### Trans-cancer migration assay

E0771-MOCK or E0771-SULT cells (8 × 10^4^ cells) were loaded on the Matrigel basement membrane matrix in the upper chamber (354480, Corning, NY, USA) and incubated at 37°C for 5 h. After the medium in the lower chamber was replaced with complete RPMI medium containing CCL21 (200 ng ml^−1^), splenocytes (1 × 10^6^) were added to the top chamber. After 2 h culture at 37°C, cells in the lower chamber were collected and stained with phycoerythrin (PE)-conjugated anti-mouse CD8a antibody and allophycocyanin (APC)-conjugated anti-mouse CD4 antibody. The assays were done on a FACSVerse™ (BD Biosciences, San Jose, CA, USA).

### T-cell isolation and activation

CD4^+^ or CD8^+^ T cells were isolated from the spleen and peripheral lymph nodes of OTII (with or without DOCK2 expression) and OTI TCR transgenic mice by magnetic sorting with Dynabeads mouse CD4 (Invitrogen) followed by treatment with DETACHaBEAD™ mouse CD4 (Invitrogen), or by a CD8a^+^ T Cell Isolation Kit (Miltenyi Biotec, Bergisch Gladbach, Germany), respectively. For antigen stimulation, OTII CD4^+^ T cells or OTI CD8^+^ T cells (1 × 10^6^ cells per well) were cultured for 2–3 days in a 24-well plate with T cell depleted, irradiated C57BL/6 spleen cells (3–5 × 10^6^ cells per well) in a total volume of 2 ml in the presence of OVA323–339 (0.5 µg ml^−1^) or OVA257–264 (0.5 µg ml^−1^), respectively. Viable cells were recovered by density gradient centrifugation using the Lympholyte-M cell separation media (Cedarlane, Hornby, Ontario, Canada). The purity of live OTII CD4^+^ T cells (CD4^+^Vα2^+^Vβ5^+^) or OTI CD8^+^ T cells (CD8a^+^Vα2^+^Vβ5^+^) was above 98%, as assessed by flow cytometry.

### Transplantation and in vivo tumor growth

After washing with phosphate-buffered saline (PBS), the following numbers of cancer cells were re-suspended in 200 µl of PBS and injected into the back of 6- to 8-week-old female C57BL/6 mice, *Sult2b1*^*–/–*^ mice or BALB/c nude mice: E0771-SULT and E0771-MOCK (1 × 10^6^ per mouse), E0771-SULT-PD-L1 and E0771-MOCK-PD-L1 (1 × 10^6^ per mouse), E0771-SULT-I-A^b^/OVA and E0771-control-I-A^b^/OVA (2 × 10^6^ per mouse), Pan02-control and Pan02-ΔSULT (1 × 10^6^ per mouse), 3LL-SULT-I-A^b^/OVA and 3LL-control-I-A^b^/OVA (3 × 10^6^ per mouse), E.G7-OVA-SULT and E.G7-OVA-MOCK (1 × 10^6^ per mouse), E0771-OVA-SULT and E0771-OVA-control (2 × 10^6^ per mouse), MC38-control, MC38-SULT, MC38-ΔCH25 and MC38-ΔCH25-SULT (5 × 10^5^ per mouse). Tumor growth was monitored twice a week by measuring perpendicular diameters with a caliper and calculating tumor volume according to the following formula: volume = p/6 ×√(length × width)^3^.

### Isolation of tumor-infiltrating cells

Tumors dissected from mice were collected into 15 ml polypropylene tubes containing 4 ml of Hanks’ balanced salt solution (HBSS, 14175, Gibco, Grand Island, NY, USA) supplemented with 2% FBS, collagenase (0.05 mg ml^−1^; C-2139, Sigma-Aldrich), 1 mM sodium pyruvate, 25 mM HEPES and DNase I (10 mg ml^−1^; Roche, Basel, Switzerland), and incubated in a 37°C water bath with shaking at 80 r.p.m. for 30 min. Digested tumor samples were passed through a 75-µm cell strainer, suspended in 4 ml of fresh HBSS and placed on ice to avoid further digestion before flow cytometric analyses or cytometry by time-of-flight (CyTOF) analyses. For sorting of tumor-infiltrating immune cells, tumors dissected from mice were dissociated using Tissue dissociation kits and gentleMACS Dissociators (Miltenyi Biotec). Then, tumor-infiltrating cells were layered onto a discontinuous Percoll (GE Healthcare, Chicago, IL, USA) gradient. After centrifugation, cells at the 35/70% interface were recovered and washed twice with HBSS. Cell sorting was done on a FACSMelody™ (BD Biosciences).

### Flow cytometry

The following antibodies and reagents were used: anti-Fcg III/II receptor (2.4G2, 0.5 µg ml^−1^; TONBO Biosciences, San Diego, CA, USA, for blocking), APC-conjugated anti-mouse CD274 (PD-L1; 10F.9G2, 2 µg ml^−1^; Biolegend, San Diego, CA, USA), APC-conjugated Rat IgG2b (RTK4530, 2 µg ml^−1^; Biolegend), PE-conjugated anti-MHC II (M5/114.15.2, 0.2 µg ml^−1^; Invitrogen), PE-conjugated Rat IgG2b (A95-1; 0.2 µg ml^−1^; BD Pharmingen), FITC-conjugated anti-mouse CD3ε (145-2C11, 5 µg ml^−1^; BD Pharmingen), FITC-conjugated anti-mouse CD4 (RM4-5, 5 µg ml^−1^; BD Pharmingen), PE-conjugated anti-mouse CD4 (RM4-5, 2 µg ml^−1^; BD Pharmingen), PE-conjugated anti-mouse CD8a (53-6.7, 2 µg ml^−1^; TONBO Biosciences), PerCP-Cy5.5-conjugated anti-mouse CD8a (2.43, 2 µg ml^−1^; TONBO Biosciences), PerCP-Cy5.5-conjugated anti-mouse Vα2 (B20.1, 2 µg ml^−1^; BD Pharmingen), biotin-conjugated anti-mouse Vβ5 (MR9-4, 5 µg ml^−1^; BD Pharmingen), APC-streptavidin (0.4 µg ml^−1^; BD Pharmingen), PE-conjugated anti-mouse CD366 (TIM-3; 5D12, 2 µg ml^−1^; BD Pharmingen), PerCP-Cy5.5-conjugated anti-mouse CD45 (30-F11, 2 µg ml^−1^; Biolegend), APC-conjugated anti-mouse CD279 [programmed cell death 1 (PD-1); RMP1-30, 2 µg ml^−1^; Biolegend], biotin-conjugated anti-mouse F4/80 (BM8.1, 5 µg ml^−1^; TONBO Biosciences), PE-Cy7-conjugated anti-mouse CD3ε (145-2C11, 2 µg ml^−1^; BD Pharmingen) and PE-Cy7-conjugated anti-mouse CD45 (30-F11, 2 µg ml^−1^; Biolegend). Flow cytometry was performed on a FACSCalibur™ or FACSVerse™ cytometer (BD Biosciences).

### CyTOF analyses

Tumor-infiltrating leukocytes were suspended in PBS with 1 µM Cell-ID Cisplatin 194Pt (Fluidigm, South San Francisco, CA, USA) in a 15 ml polypropylene tube, and incubated for 5 min at room temperature before quenching with MaxPar Cell Staining buffer (Fluidigm). Cells were centrifuged at 500 × *g* for 7 min at 4°C, re-suspended in 50 µl MaxPar Cell Staining buffer with Fc block (CD16/32; BD Pharmingen) and incubated for 10 min at room temperature. Then, cells were reacted with metal-tagged cell-surface antibodies (Fluidigm) for another 30 min at room temperature, and washed with MaxPar Cell Staining buffer at 500 × *g* for 7 min at 4°C. For further staining for intracellular proteins, cells were fixed in 1 ml fresh 1× Maxpar Fix I buffer (Fluidigm) for 15 min at room temperature, washed with 4 ml MaxPar Perm-S buffer (Fluidigm) at 800 × *g* for 7 min at 4°C and re-suspended in 50 µl MaxPar Perm-S buffer with metal-tagged cytoplasmic/secreted antibodies (Fluidigm). After incubation for 30 min at room temperature, cells were washed with MaxPar Cell Staining buffer. Samples were re-suspended in 1 ml of MaxPar Fix and Perm buffer (Fluidigm) with 125 nM Cell-ID Intercalator-Ir (Fluidigm) overnight at 4°C. The next day, cells were washed twice with 2 ml MaxPar Cell Staining buffer, and re-suspended in MaxPar Cell Acquisition Solution (Fluidigm). Cells were passed through a 35-µm strainer (Falcon) twice to eliminate the clumped cells before analyzing by CyTOF Mass Cytometers (Helios; Fluidigm). EQ Four Element Calibration Beads (Fluidigm) were used to normalize signal intensity over time with the CyTOF software program (Fluidigm). Acquired data were uploaded to a Cytobank web server 8.0 (Cytobank, Santa Clara, CA, USA) for data processing and gating out of dead cells, doublets and normalization beads. The antibody information is listed in Supplementary Table S1.

### Matrix-assisted laser desorption/ionization imaging mass spectrometry

Matrix-assisted laser desorption/ionization (MALDI) imaging analyses were performed as described previously (21), using an Ultraflextreme MALDI-TOF/TOF mass spectrometer and 7T FT-ICR-MS (Solarix Bruker Daltonik) equipped with an Nd:YAG laser. Data were acquired in the negative reflectron mode with raster scanning by a pitch distance of 50 µm. Each spectrum was the result of 300 laser shots at each data point. In the TOF/TOF measurement, signals between m/z 50 and 1000 were collected. In FT-ICR-MS imaging, signals between m/z 300 and 500 were collected by continuous accumulation of selected ion mode. Image reconstruction for both was performed using the FlexImaging 4.1 software program (Bruker Daltonics). Molecular identification was performed using FT-ICR-MS data; the high mass accuracy provided by FT-ICR-MS allowed selective ion signals for the metabolites to be obtained within a mass window of 5 p.p.m., enabling the identification of the specific elemental composition of compounds by querying the highly accurate masses against database.

### Immunohistochemistry

For clinical specimens, tissue samples were fixed in neutral buffer solution containing 10% formalin for 5 min and incubated in ethylenediaminetetraacetic acid (EDTA) buffer (pH 9.0) for 10 min. After inactivation of endogenous peroxidase with 0.3% H_2_O_2_ in methanol for 15 min, the sections were first incubated with anti-human CD8 (C8/144B, 1:100; Nichirei Biosciences Inc., Tokyo, Japan), then reacted with ImmPRESS® (anti-mouse IgG polymer detection kit; Vector Laboratories, Burlingame, CA, USA) and visualized with DAB buffer tablets (Muto Pure Chemicals Co., Ltd., Tokyo, Japan).

### Quantification of CS and oxysterols by mass spectrometry

The amounts of CS in human cancer tissue samples were quantified as described previously (21). To monitor the production of CS and/or oxysterols in E0771, 3LL, E.G7-OVA and MC38 cells with or without the expression of SULT2B1b, cells were seeded at 5 × 10^5^ cells per well in the 6-well plates, and the culture supernatants and cell pellets were collected 24 h later. CS and oxysterols were extracted from cellular pellets or cell culture supernatant, respectively, by 1 ml of extraction solvent (methanol/chloroform/water, 5:2:2, *v/v/v*) after supplementing of deuterium-labeled internal standard (IS) compounds (d7-CS; d7-cholesterol; and d6-25-HC). Samples were mixed vigorously by vortex for 1 min followed by 5 min of sonication. Then, the samples were centrifuged at 16 000 × *g* for 5 min at 4°C, and the supernatant (700 µl) was collected into clean tubes. After mixing with 235 µl of chloroform and 155 µl of water, the aqueous and organic layers were separated by vortex, followed by centrifugation. The 200 µl of bottom layer was dried under a stream of nitrogen. For the liquid chromatography tandem mass spectrometry (LC/MS/MS)-based quantitative analysis of CS and oxysterols, the dried samples were reconstituted in 50 µl of methanol. A triple-quadrupole mass spectrometer equipped with an electrospray ionization (ESI) ion source (LCMS-8060; Shimadzu Corporation) was used in multiple reaction monitoring (MRM) mode. The conditions for the LC/MS/MS analysis were as follows: column, Inertsil ODS-4 (2.1 × 150 mm; particle size, 3 µm; GL Sciences Inc.); column temperature, 40°C; flow rate, 0.3 ml min^−1^; mobile phase, 5 mM ammonium acetate in water/acetonitrile (1:2, *v/v*) (A) and 5 mM ammonium acetate in methanol/isopropanol (1:19, *v/v*) (B); gradient curve, 0% B at 0 min, 100% B at 22 min, 100% B at 27 min, 0% B at 27.1 min, and 0% B at 30 min; injection volume, 5 µl; mass analysis mode, positive and negative ion mode; electrospray voltage, 4 kV in the positive ion mode and ‒3.5 kV in the negative ion mode; nebulizer gas flow rate, 3.0 L min^−1^; drying gas flow rate, 10.0 L min^−1^; desolvation temperature, 250°C; heat block temperature, 400°C; and detector voltage, 2.16 kV. The MRM mode and a dwell time of 2 ms per channel were used. The absolute contents of CS and oxysterols were calculated based on the calibration curves. The MRM calibration curves were generated from the triplicate analyses of these standard solutions using the chromatographic peak area of each analyte to that of the IS.

### Quantification and statistical analysis

Statistical analyses were performed using Prism 7.0 (GraphPad Software, San Diego, CA, USA). The data were initially tested for normality using the Kolmogorov–Smirnov test. Parametric data were analyzed using a two-tailed unpaired Student’s *t-*test when two groups were compared. Nonparametric data were analyzed with a two-tailed Mann–Whitney test when two groups were compared. Statistical differences between more than two experimental groups were evaluated using an analysis of variance (ANOVA) with Dunnett’s *post hoc* test. The *P*-value of Kaplan–Meier curves was determined using the log-rank test. *P*-values less than 0.05 were considered statistically significant.

## Results

### CS production and CD8^+^ T-cell infiltration are inversely correlated in human colon cancer tissues

**Fig. 1.**
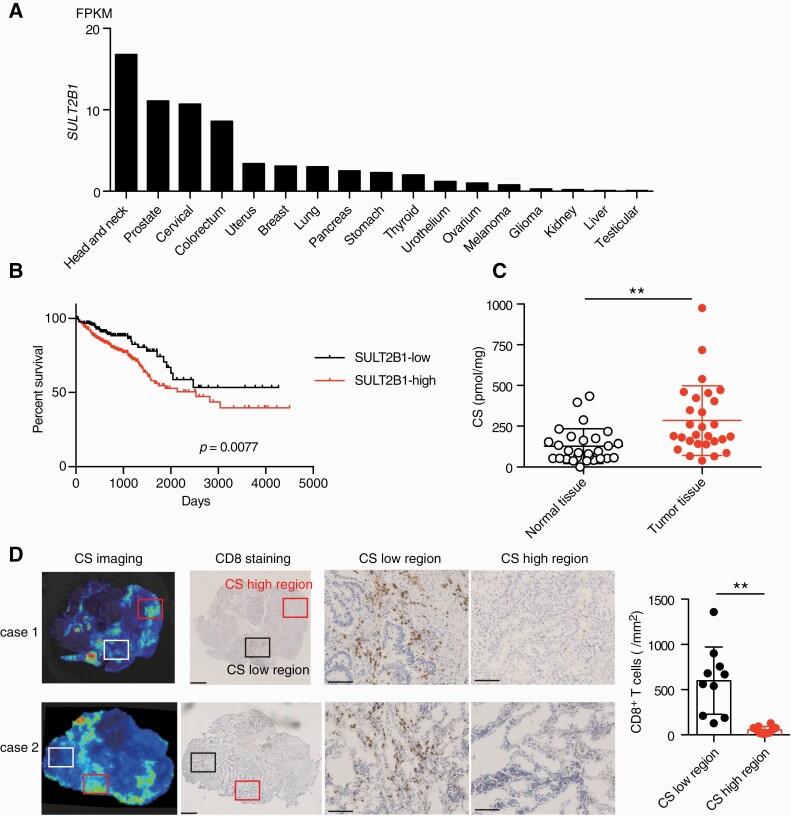
CS production and CD8^+^ T-cell infiltration are inversely correlated in colon cancer tissue samples. (A) Comparison of the *SULT2B1* expression in 17 human cancer types. Data are from the Human Protein Atlas (https://www.proteinatlas.org) and are indicated as medians of the fragments per kilobase million (FPKM) values. (B) Kaplan–Meier curves showing survival of colon cancer patients with high (*n* = 404) or low (*n* = 193) SULT2B1 expression. The cut-off value was set at 5.68. *P =* 0.0077 (log-rank test). (C) Comparison of CS production between normal colon tissues and colon cancer tissues. Data are presented as the mean ± SD. **P < 0.01 (two-tailed Mann–Whitney test). (D) Representative images for CS production and CD8^+^ T-cell infiltration in colon cancer tissue samples. Scale bars indicate 1 mm (the second column) and 250 µm (the third and fourth columns). The graph shows the comparison of the number of infiltrating CD8^+^ T cells between the CS-high and CS-low regions (*n* = 10). Data are presented as the mean ± SD. **P < 0.01 (two-tailed unpaired Student’s *t*-test).

The public database of the Human Protein Atlas (https://www.proteinatlas.org/ENSG00000088002-SULT2B1/pathology) indicates that many types of tumors, including colon cancers, highly express *SULT2B1* (Fig. 1A). In contrast, the expression of *DOCK2* is very low in all samples tested, and there is no association between expression levels of *SULT2B1* and *DOCK2* (Supplementary Figure 1), suggesting that high levels of *SULT2B1* expression are mainly attributed to its expression in cancerous tissues. When the clinical course was compared between colon cancer patients with high and low SULT2B1 expression, SULT2B1-high patients showed poor prognosis (Fig. 1B). Quantitative mass spectrometric analyses also revealed that CS was abundantly produced in colon cancer tissues, compared with that in normal colon tissues (Fig. 1C). Importantly, the CS levels in the same specimen from the colon cancer tissues (see CS-high region and CS-low region) were inversely correlated with infiltration of CD8^+^ T cells (Fig. 1D). From these results, we hypothesized that cancer-derived CS may be involved in tumor immune evasion.

### CS production in tumors inhibits infiltration by effector T cells

**Fig. 2.**
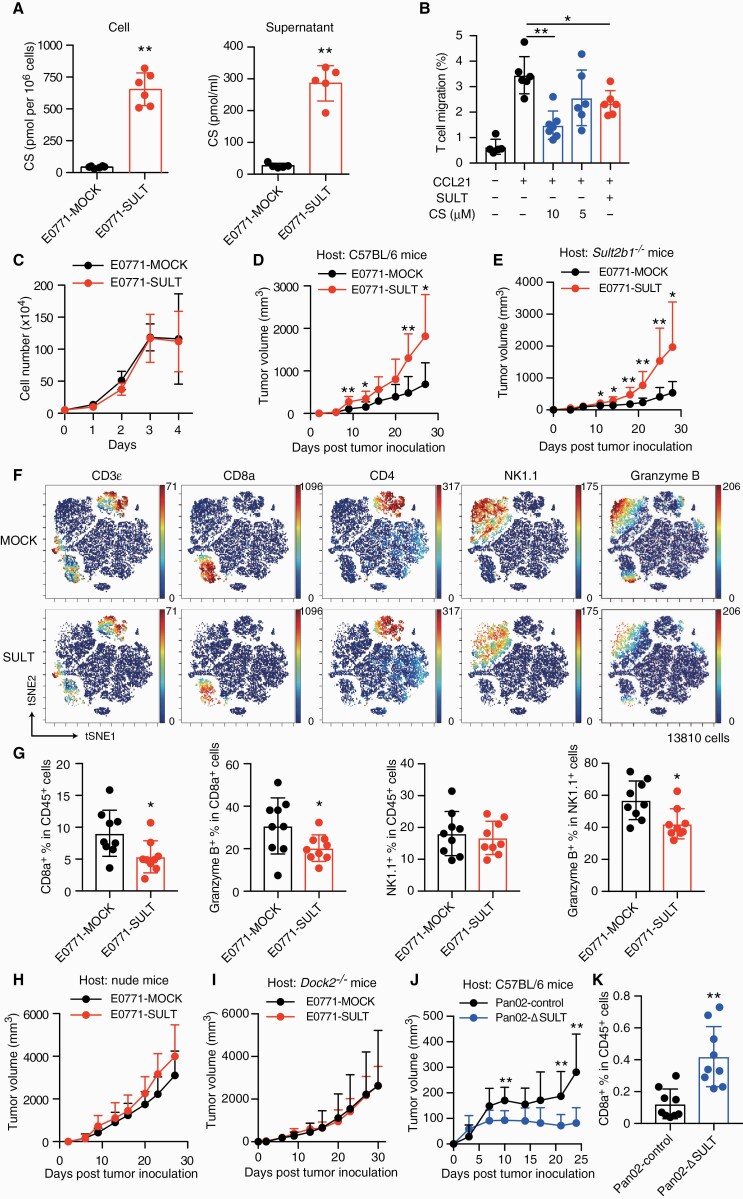
CS production in tumors inhibits infiltration of effector T cells. (A) CS production in cells (*n* = 6) and culture supernatant (*n* = 5) were compared between E0771-SULT and E0771-MOCK. Data are presented as the mean ± SD. **P < 0.01 (two-tailed unpaired Student’s *t-*test). (B) Trans-cancer migration assays showing that the presence of E0771-SULT (SULT+) and CS-treated E0771-MOCK (SULT–) in Matrigel reduces CCL21-induced T-cell migration (*n* = 5–7). Data are presented as the mean ± SD. *P < 0.05, **P < 0.01 (one-way ANOVA followed by Dunnett’s post hoc test). (C) *In vitro* growth was compared between E0771-MOCK and E0771-SULT (*n* = 5). (D and E) After transplantation, tumor growth in wild-type (WT) or *Sult2b1*^*^–/–^*^ C57BL/6 mice was compared between E0771-MOCK and E0771-SULT (*n* = 8–9). Data are presented as the mean + SD. *P < 0.05, **P < 0.01 (two-tailed unpaired Student’s *t*-test). (F) viSNE plots highlighting the distribution of tumor-infiltrating lymphocytes in E0771-SULT and E0771-MOCK. (G) The percentages of CD8^+^ T cells and NK cells in infiltrating leukocytes and their expression of granzyme B were compared between E0771-SULT and E0771-MOCK (*n* = 9). Data are presented as the mean ± SD. *P < 0.05 (two-tailed unpaired Student’s *t*-test). (H and I) After transplantation, tumor growth in BALB/c nude mice (H; *n* = 7) or DOCK2^–/–^ mice (I; *n* = 6) was compared between E0771-SULT and E0771-MOCK. (J) After transplantation, tumor growth in C57BL/6 mice was compared between Pan02-control and Pan02-ΔSULT (*n* = 10, 8). Data are presented as the mean + SD. **P < 0.01 (two-tailed Mann–Whitney test). (K) The percentages of CD8^+^ T cells in infiltrating leukocytes were compared between Pan02-control and Pan02-ΔSULT (*n* = 9). Data are presented as the mean ± SD. **P < 0.01 (two-tailed unpaired Student’s *t*-test).

To functionally examine the role of cancer-derived CS in tumor immune evasion, we retrovirally expressed SULT2B1b in a mouse breast cancer cell line E0771, which lacks its endogenous expression (Supplementary Figure 2A). In comparison to E0771-MOCK expressing the vector alone (pMX-IRES-GFP), this cell line (designated E0771-SULT) not only produced, but also secreted, large amounts of CS (Fig. 2A). In ‘trans-cancer’ migration assays, the presence of E0771-SULT cells in Matrigel significantly reduced CCL21-induced T-cell migration as compared with that of E0771-MOCK (Fig. 2B). Similar results were obtained when E0771-MOCK cells were treated with CS (Fig. 2B), indicating that CS mediates T-cell exclusion.

Although the expression of SULT2B1b did not affect the growth of E0771 cells *in vitro* (Fig. 2C), the growth of E0771-SULT cells was much faster than E0771-MOCK cells when transplanted into syngeneic C57BL/6 mice (Fig. 2D). Similar results were obtained when *Sult2b1*-deficient (*Sult2b1*^*–/–*^) C57BL/6 mice were used as recipients (Fig. 2E). A CyTOF analysis revealed that infiltrating CD8^+^ T cells and their granzyme expression were reduced in the case of E0771-SULT cells (Fig. 2F and G), suggesting that cancer-derived CS suppresses anti-tumor T-cell responses, most likely by inhibiting the Rac GEF activity of DOCK2 (21). In support of this, the difference in tumor growth between E0771-SULT and E0771-MOCK disappeared when transplanted into nude mice lacking T cells (Fig. 2H) or DOCK2^–/–^ mice (Fig. 2I). Although the expression of *Sult2b1b* in E0771-SULT cells was higher than that in human colon cancer tissues, a mouse pancreas cancer cell line Pan02 showed comparable expression (Supplementary Figure 3). We also found that genetic deletion of *Sult2b1* in Pan02 (Pan02-ΔSULT) suppressed tumor growth in C57BL/6 mice (Fig. 2J; Supplementary Figure 2B), with an increased percentage of tumor-infiltrating CD8^+^ T cells (Fig. 2K).

### CS production renders tumor cells resistant to immune checkpoint blockade

**Fig. 3.**
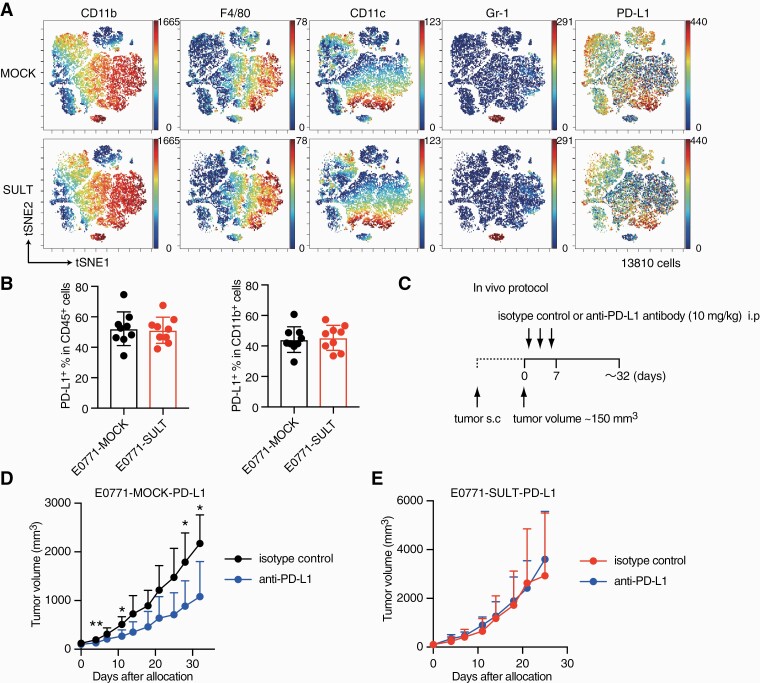
CS production renders tumor cells resistant to immune checkpoint blockade. (A) viSNE plots highlighting the distribution of tumor-infiltrating myeloid cells in E0771-MOCK and E0771-SULT. (B) The percentage of PD-L1^+^ cells in infiltrating leukocytes or CD11b^+^ cells was compared between E0771-MOCK and E0771-SULT (*n* = 9). Data are presented as the mean ± SD. (C) Schematic illustration of the protocol used for anti-PD-L1 treatment. (D and E) After transplantation, tumor growth of E0771-MOCK-PD-L1 (D, *n* = 5) or E0771-SULT-PD-L1 (E, *n* = 6) was compared between mice treated with anti-PD-L1 antibody and those treated with isotype-matched control. Data are presented as the mean + SD. *P < 0.05, **P < 0.01 (two-tailed Mann–Whitney test).

The interaction between PD-1 and PD-L1 is one of the most prominent targets for immune checkpoint blockade (1). However, the expression of PD-L1 on immune cells, including CD11b^+^ antigen-presenting cells was unchanged between C57BL/6 mice bearing E0771-SULT and those bearing E0771-MOCK (Fig. 3A and B). In addition, PD-1 and Tim3, markers for exhausted T cells, were comparably expressed on tumor-infiltrating T cells, splenic T cells and lymph node T cells from these mice (Supplementary Figure 4). The expression of PD-L1 was low on E0771-MOCK cells, even after treatment with IFN-γ (Supplementary Figure 5), and anti-PD-L1 antibody treatment only marginally affected growth of E0771-MOCK in C57BL/6 mice (Supplementary Figure 6). To clearly examine the effect of CS production on immune checkpoint blockade, we forced the expression of PD-L1 on E0771-SULT and E0771-MOCK cells (E0771-SULT-PD-L1 and E0771-MOCK-PD-L1), transplanted them into C57BL/6 mice, and started the antibody therapy when the tumor size reached around 150 mm^3^ (Fig. 3C). As a result, in comparison to the isotype-matched control, anti-PD-L1 antibody significantly inhibited *in vivo* growth of E0771-MOCK-PD-L1 cells in C57BL/6 mice (Fig. 3D). In contrast, E0771-SULT-PD-L1 cells did not show any response to anti-PD-L1 antibody therapy (Fig. 3E) in spite of the comparable expression of PD-L1 on their surface (Supplementary Figure 5). Thus, CS production renders cancer cells resistant to immune checkpoint blockade.

### CS-producing cancer cells exhibit resistance to cancer-specific T-cell transfer

**Fig. 4.**
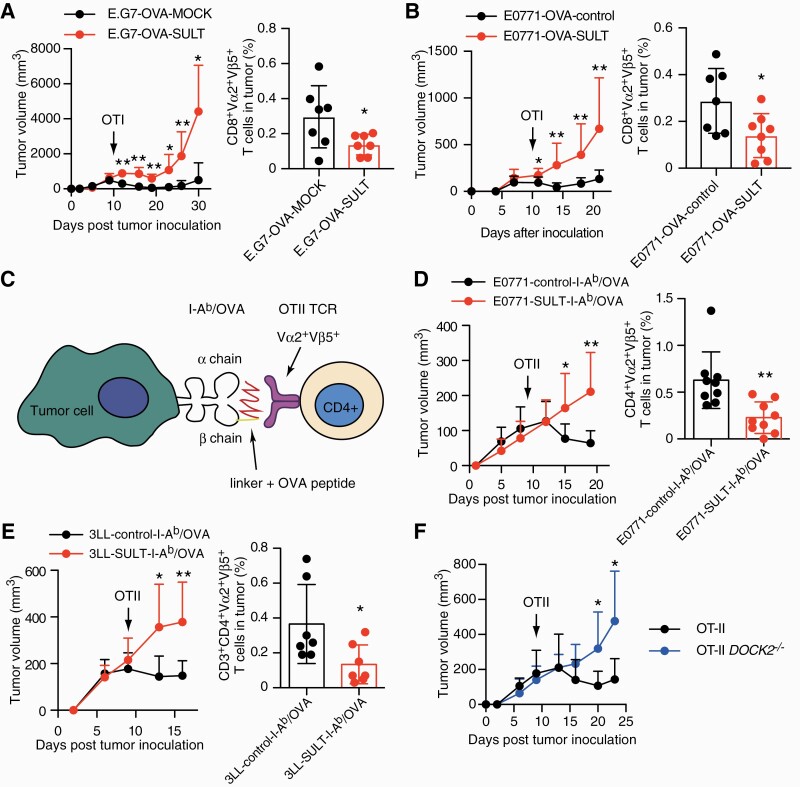
SULT2B1b-mediated CS production in tumors dampens anti-tumor T-cell responses. (A) The effect of OTI CD8^+^ T-cell transfer on tumor growth in C57BL/6 mice was compared between E.G7-OVA-MOCK (*n* = 5) and E.G7-OVA-SULT (*n* = 6). After the intravenous injection of activated Vα2^+^Vβ5^+^ OTI T cells on day 10, the infiltration of OTI T cells was analyzed on day 15 (*n* = 7). Data are presented as the mean + SD or ± SD. *P < 0.05, **P < 0.01 (two-tailed Mann–Whitney test for both tumor growth and infiltration of OTI T cells). (B) The effect of OTI CD8^+^ T-cell transfer on tumor growth in C57BL/6 mice was compared between E0771-OVA-control (*n* = 8) and E0771-OVA-SULT (*n* = 8). After the intravenous injection of activated Vα2^+^Vβ5^+^ OTI T cells on day 10, the infiltration of OTI T cells was analyzed on day 15 (*n* = 7 or 8). Data are presented as the mean + SD or ± SD. *P < 0.05, **P < 0.01 (two-tailed Mann–Whitney test for tumor growth and two-tailed unpaired Student’s *t-*test for infiltration of OTI T cells). (C) Schematic representation of the interaction between OTII CD4^+^ T cells and tumor cells expressing I-A^b^ molecule covalently bound to OVA peptide. (D and E) The effect of OTII CD4^+^ T-cell transfer on tumor growth in C57BL/6 mice was compared between (D) E0771-control-I-A^b^/OVA (*n* = 8) and E0771-SULT-I-A^b^/OVA (*n* = 7) or (E) 3LL-control-I-A^b^/OVA (*n* = 8) and 3LL-SULT-I-A^b^/OVA (*n* = 8). After the intravenous injection of activated Vα2^+^Vβ5^+^ OTII T cells on day 9, infiltration of OTII T cells was analyzed on day 14 (D, *n* = 9; E, *n* = 7). Data are presented as the mean + SD or ± SD. *P < 0.05, **P < 0.01 (two-tailed unpaired Student’s *t-*test for tumor growth and two-tailed Mann–Whitney test for infiltration of OTII T cells). (F) Comparison of the effect of DOCK2^+/+^ OTII CD4^+^ T cells and DOCK2^–/–^ OTII CD4^+^ T cells (*n* = 7) on the growth of E0771-control-I-A^b^/OVA transplanted into C57BL/6 mice. Data are presented as the mean + SD. *P < 0.05 (two-tailed unpaired Student’s *t-*test).

To examine the role of CS in cancer-specific CD8^+^ T-cell responses, we used E.G7-OVA (29), which was developed by introducing the gene encoding full-length OVA into a mouse T-cell lymphoma cell line, EL4. In E.G7-OVA, through antigen processing of OVA protein, OVA257–264 peptide is presented by the MHC class I H-2K^b^ molecule on their surface (29), which is recognized by CD8^+^ T cells from OTI TCR transgenic mice (30). When CD8^+^ T cells from OTI TCR transgenic mice were activated *in vitro* and injected intravenously into C57BL/6 mice on day 10 after transplantation of E.G7-OVA-MOCK, tumor progression was suppressed (Fig. 4A). However, E.G7-OVA cells expressing SULT2B1b (E.G7-OVA-SULT) exhibited resistance to this therapy, with a lower percentage of tumor-infiltrating OTI CD8^+^ T cells (Fig. 4A and Supplementary Figure 2A). Similar results were obtained when the same protocol was applied to E0771 cells expressing OVA protein with or without the expression of SULT2B1b (E0771-OVA-SULT and E0771-OVA-control) (Fig. 4B).

Recent evidence indicates that CD4^+^ T cells can target tumor cells in various ways, either directly by eliminating tumor cells or indirectly by modulating the tumor microenvironment (31, 32). To examine the role of cancer-derived CS on tumor-specific CD4^+^ T-cell responses, we expressed the I-A^b^ molecule covalently bound to OVA323–339 (I-A^b^/OVA complex; Fig. 4C) as an ‘artificial tumor-specific antigen’ on E0771 and 3LL with or without Sult2B1b expression (E0771-SULT-I-A^b^/OVA; 3LL-SULT-I-A^b^/OVA; E0771-control-I-A^b^/OVA; 3LL-control-I-A^b^/OVA) (Supplementary Figures 2A and 7). OTII TCR recognizes OVA peptide (OVA323–339) presented by MHC class II I-A^b^ molecules (33). When CD4^+^ T cells from OTII TCR transgenic mice were activated *in vitro* and injected intravenously into C57BL/6 mice on day 9 after transplantation of E0771-control-I-A^b^/OVA or 3LL-control-I-A^b^/OVA, tumor growth was markedly suppressed (Fig. 4D and E). However, the adoptive transfer of OTII CD4^+^ T cells failed to inhibit tumor progression when they expressed SULT2B1b (Fig. 4D and E). Similarly, this inhibitory effect was lost when DOCK2-deficient (DOCK2^–/–^) OTII CD4^+^ T cells were administered (Fig. 4F). Collectively, these results indicate that CS production by tumor cells dampens anti-tumor T-cell responses by inhibiting the function of DOCK2.

### The opposite effect of the expression of SULT2B1b on anti-tumor responses of oxysterol-producing tumors and oxysterol-non-producing tumors

**Fig. 5.**
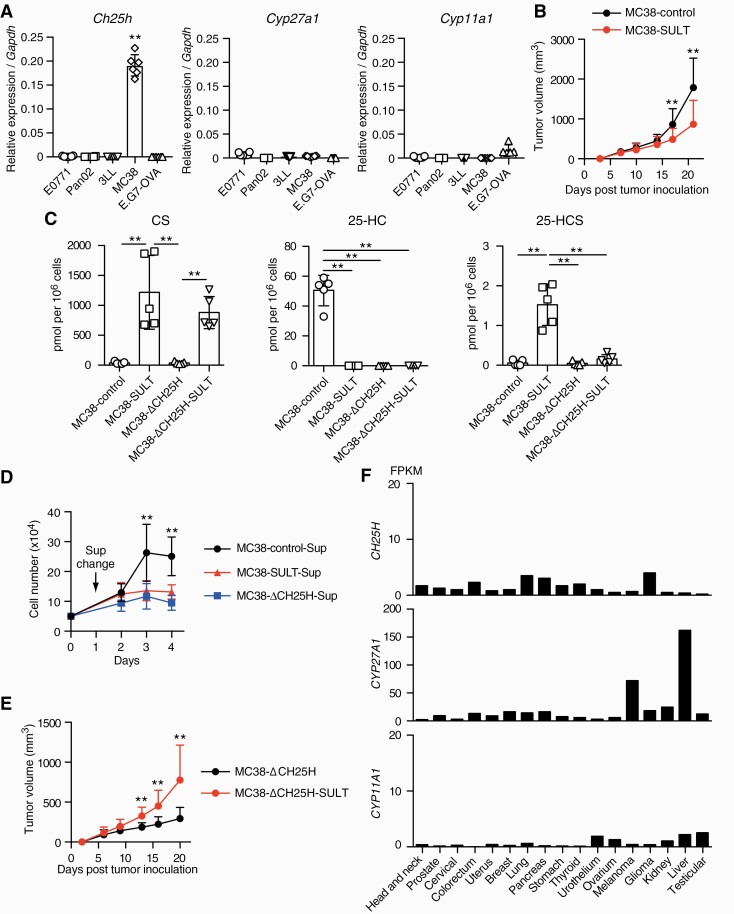
The expression of SULT2B1b suppresses *in vivo* growth of MC38 cells that produce 25-HC. (A) The expression of *Ch25h*, *Cyp27a1* and *Cyp11a*1 in tumor cell lines. Reverse transcription-quantitative PCR showing high expression of Ch25h in MC38. Data (*n* = 5–7) are presented as the mean ± SD after normalization with the *Gapdh* expression. **P < 0.01 (one-way ANOVA followed by Dunnett’s post hoc test). (B) After transplantation, tumor growth in C57BL/6 mice was compared between MC38-SULT and MC38-control (*n* = 10). Data are presented as the mean + SD. **P < 0.01 (two-tailed Mann–Whitney test). (C) Production of CS, 25-HC and 25-HCS in MC38-control, MC38-SULT, MC38-ΔCH25 and MC38-ΔCH25-SULT (*n* = 5 per each) was quantified by mass spectrometry. Data are presented as the mean ± SD. **P < 0.01 (one-way ANOVA followed by Dunnett’s post hoc test). (D) *In vitro* growth of MC38 was analyzed in the presence of the culture supernatant from wild-type MC38, MC38-SULT or MC38-ΔCH25 (*n* = 6 per each). Data are presented as the mean ± SD. **P < 0.01 (one-way ANOVA followed by Dunnett’s post hoc test). (E) After transplantation, tumor growth in C57BL/6 mice was compared between MC38-ΔCH25 (*n* = 8) and MC38-ΔCH25-SULT (*n* = 9). Data are presented as the mean + SD. **P < 0.01 (two-tailed unpaired Student’s *t-*test). (F) The expression of genes encoding oxysterol hydroxylases in 17 human cancer types. Data are from the Human Protein Atlas (https://www.proteinatlas.org) and are indicated as medians of the fragments per kilobase per million (FPKM) values.

Oxysterols such as 25-HC are oxidized derivatives of cholesterol that regulate the expression of a wide range of metabolic and inflammatory genes through liver X receptor (LXR) signaling (34–36). In addition, oxysterols have been shown to favor tumor growth directly, by promoting tumor cell growth, and indirectly, by dampening anti-tumor immune responses (25–27). Unlike E0771, Pan02, E.G7-OVA and 3LL, the mouse colon cancer cell line MC38 highly expressed CH25H (Fig. 5A), which is responsible for the conversion of cholesterol to 25-HC (37). Although CH25H is up-regulated in immune cells that are exposed to inflammatory mediators (38), the expression levels of CH25H in tumor-infiltrating macrophages and T cells were much lower in comparison to the level in MC38 cells (Supplementary Figure 8). Interestingly, we found that the expression of SULT2B1b in MC38 cells (MC38-SULT) did not promote—rather it inhibited—tumor progression *in vivo* (Fig. 5B). It is known that SULT2B1b sulfates oxysterols and inactivates them (24–27, 36). Consistent with this, quantitative mass spectrometric analyses revealed that in MC38-SULT cells, the production of 25-HC was lost, but the level of 25-HCS markedly increased (Fig. 5C). To examine whether 25-HC affects tumor growth, we treated MC38 cells with culture supernatants from various MC38-derivatives. Although the supernatant from wild-type MC38 cells promoted the cell growth of MC38 cells *in vitro*, this effect was not seen for those from MC38-SULT and MC38-ΔCH25H lacking the expression of CH25H (Fig. 5D), suggesting the relevance of 25-HC to tumor growth. Consistent with this, direct stimulation of MC38-ΔCH25H with 25-HC, but not 25-HCS, promoted tumor growth *in vitro* (Supplementary Figure 9). Importantly, we found that the expression of SULT2B1b in MC38-ΔCH25H cells significantly augmented tumor progression when transplanted into C57BL/6 mice (Fig. 5E). In addition, the Human Protein Atlas also indicates that the expression of cholesterol hydroxylases is relatively limited to certain tumor types, such as hepatocellular carcinoma and melanoma, and is low in *Sult2b1*-expressing cancers (Fig. 5F). Thus, SULT2B1b inhibition could be a therapeutic strategy to disrupt tumor immune evasion in many cancers.

## Discussion

An important question in tumor immunology is why many patients exhibit resistance to immunotherapies. Although accumulating evidence indicates that resistance to immunotherapy is associated with a decrease in T-cell infiltration into tumors, the underlying mechanisms are still poorly understood. We identified cancer-derived CS as a key mediator of T-cell exclusion in the tumor microenvironment. By analyzing clinical samples, we found that the levels of CS were inversely correlated with the infiltration of CD8^+^ T cells in human colon cancer tissues. CS is a cell-active inhibitor of DOCK2, a Rac GEF critical for migration and activation of lymphocytes (21). Consistent with this, treatment of E0771-MOCK with CS inhibited CCL21-induced T-cell migration *in vitro* in trans-cancer migration assays. In addition, the cancer cell lines E0771, 3LL and E.G7-OVA engineered to produce CS exhibited resistance to cancer-specific T-cell transfer and immune checkpoint blockade *in vivo*, with a reduction of infiltrating T cells. Thus, CS constitutes a ‘chemical barrier’ to prevent tumor infiltration by effector T cells in several SULT2B1b-expressing tumor models.

Paradoxically, however, the expression of SULT2B1b in MC38 cells did not promote—rather it inhibited—tumor progression. Since the tumor-promoting activity of SULT2B1b could be recovered in MC38-ΔCH25H, it is clear that the production of 25-HC is involved in this process. SULT2B1b mediates CS production, but its other known substrates are oxysterols, such as 25-HC (24–27, 36). In this study, we have shown that 25-HC promoted growth of MC38 cells *in vitro*, whereas 25-HCS failed to do so. In addition, it has been reported that 25-HC, but not 25-HCS, impairs T-cell priming by inhibiting migration of dendritic cells (DCs) to the draining lymph nodes (26). Therefore, in tumors expressing cholesterol hydroxylases, the expression of SULT2B1b would lead to anti-tumor responses by inactivating oxysterols, whereas in tumors lacking cholesterol hydroxylases, the expression of SULT2B1b would dampen anti-tumor T-cell response by inducing tumor immune evasion. Importantly, in humans, the expression of cholesterol hydroxylases is relatively limited to certain tumor types and is low in *Sult2b1*-expressing cancers. Therefore, SULT2B1b could be a therapeutic target for promoting anti-tumor immunity in many cancers.

In conclusion, we have shown that CS-producing cancer cells exhibit resistance to cancer-specific T-cell transfer and immune checkpoint blockade unless they produce oxysterols. Our findings define a previously unknown mechanism of tumor immune evasion and provide a novel insight into the development of effective immunotherapies.

## Supplementary Material

dxac002_suppl_Supplementary_Material
