# Supplementary material for: Cancer-derived cholesterol sulfate is a key mediator to prevent tumor infiltration by effector T cells
Source: Int Immunol. 2022 Jan 30;34(5):277–89. doi: 10.1093/intimm/dxac002 (PMC9020568; doi:10.1093/intimm/dxac002)
Supplement: dxac002_suppl_Supplementary_Material [file dxac002_suppl_supplementary_material.pdf]

Supplementary Fig. 1. The expression of *DOCK2* in 17 human cancer tissues. Data are from the Human Protein Atlas (https://www.proteinatlas.org) and are indicated as medians of the FPKM values.

Supplementary Fig. 2. The expression of SULT2B1b in tumor cell lines used in the study. (A) Representative immunoblot showing SULT2B1b and actin in tumor cell lines. Total cell lysates (30 μg protein per lane) were used. Asterisks indicate SULT2B1b with (*) and without (**) HA-tag, respectively. (B) Expression of SULT2B1b in Pan02 and Pan02-ΔSULT cell lines. Total cell lysates (30 μg protein per lane) were used.

Supplementary Fig. 3. Comparison of *SULT2B1b* expression among human colon cancer tissues (n = 6) and two mouse cell lines, E0771-SULT and Pan02 (n = 3 per each cell line). Data are presented as the mean + SD. $^{**}P < 0.01$ (two-tailed Mann-Whitney test).

Supplementary Fig. 4. Comparable expression of PD-1 and Tim3 on tumor-infiltrating T cells in mice bearing E0771-MOCK and those bearing E0771-SULT. The expressions of PD-1 and Tim-3 on tumor-infiltrating T cells, splenic T cells and lymph node T cells were analyzed on day 21 after transplantation of E0771-MOCK or E0771-SULT cells. Data (n = 5-6) are expressed as mean ± SD. As a reference, tumor volume was also compared at sacrifice.

Supplementary Fig. 5. Comparison of the PD-L1 expression among E0771-MOCK, E0771-MOCK-PD-L1 and E0771-SULT-PD-L1. E0771-MOCK cells were treated with or without IFN-γ (20ng/ml) for 24 hours before assays.

Supplementary Fig. 6. No significant effect of anti-PD-L1 antibody treatment on in vivo growth of E0771-MOCK transplanted into C57BL/6 mice. Schematic illustration of the protocol used for anti-PD-L1 treatment is shown. After transplantation, tumor growth of E0771-MOCK (n = 6) or E0771-SULT-PD-L1 (n = 7) was compared between mice treated with anti-PD-L1 antibody and those treated with isotype-matched control. Data are presented as the mean + SD.

Supplementary Fig. 7. Comparable expression of I-A^b/OVA complex between E0771-control-I-A^b/OVA and E0771-SULT-I-A^b/OVA or 3LL-control-I-A^b/OVA and 3LL-SULT-I-A^b/OVA. Representative FACS profiles of cells stained with anti-I-A^b antibody (filled) or isotype-matched control (blank) are shown.

Supplementary Fig. 8. The expression of *Ch25h* in MC38 or MC38-infiltrating macrophages and T cells. Three-four weeks after transplantation of MC38 into C57BL/6 mice, tumor infiltrating CD45$^+$F4/80$^+$ macrophages and CD45$^+$CD3ε^+ T cells were sorted by fluorescence cell sorting. Data (n = 5-6) are presented as the mean ± SD after normalization with *Gapdh* expression. **P* < 0.05 (one way ANOVA followed by Dunn’s post hoc test).

Supplementary Fig. 9. 25-HC, but not 25-HCS, promotes growth of MC38-ΔCH25H *in vitro*. MC38-ΔCH25H cells were stimulated with 25-HC (10 nM), 25-HCS (10 nM) or vehicle (DMSO) alone for 3 days in D-MEM medium containing 0.3% FBS. Data (n = 5) are presented as the mean ± SD. **P* < 0.05, ***P* < 0.01 (one way ANOVA followed by Dunn’s post hoc test).

Supplementary Table 1. Mass cytometry antibodies panel design

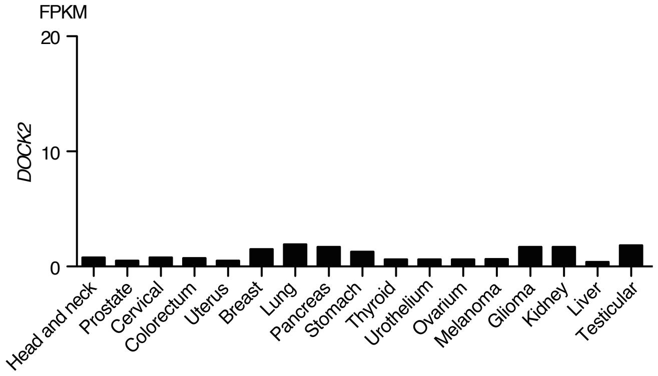
FPKM
20
10
0
DOCK2
Head and neck
Prostate
Cervical
Colorectum
Uterus
Breast
Lung
Pancreas
Stomach
Thyroid
Urothelium
Ovarium
Melanoma
Glioma
Kidney
Liver
Testicular

Fig. S1

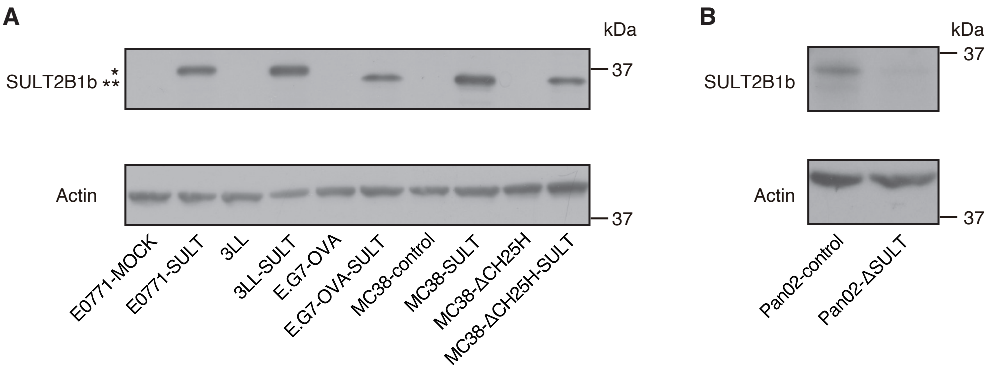
A
B
kDa
SULT2B1b * **
37
Actin
37
E0771-MOCK
E0771-SULT
3LL
3LL-SULT
E.G7-OVA
E.G7-OVA-SULT
MC38-control
MC38-SULT
MC38-ΔCH25H
MC38-ΔCH25H-SULT
kDa
SULT2B1b
37
Actin
37
Pan02-control
Pan02-ΔSULT

Fig. S2

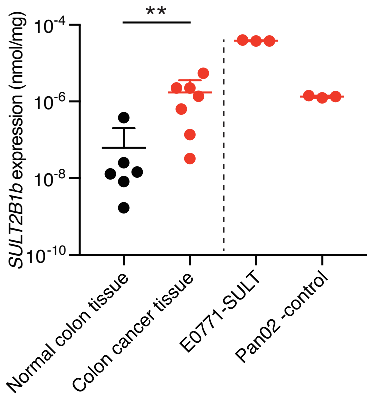
**
SULT2B1b expression (nmol/mg)
10-4
10-6
10-8
10-10
Normal colon tissue
Colon cancer tissue
E0771-SULT
Pan02 -control

Fig. S3

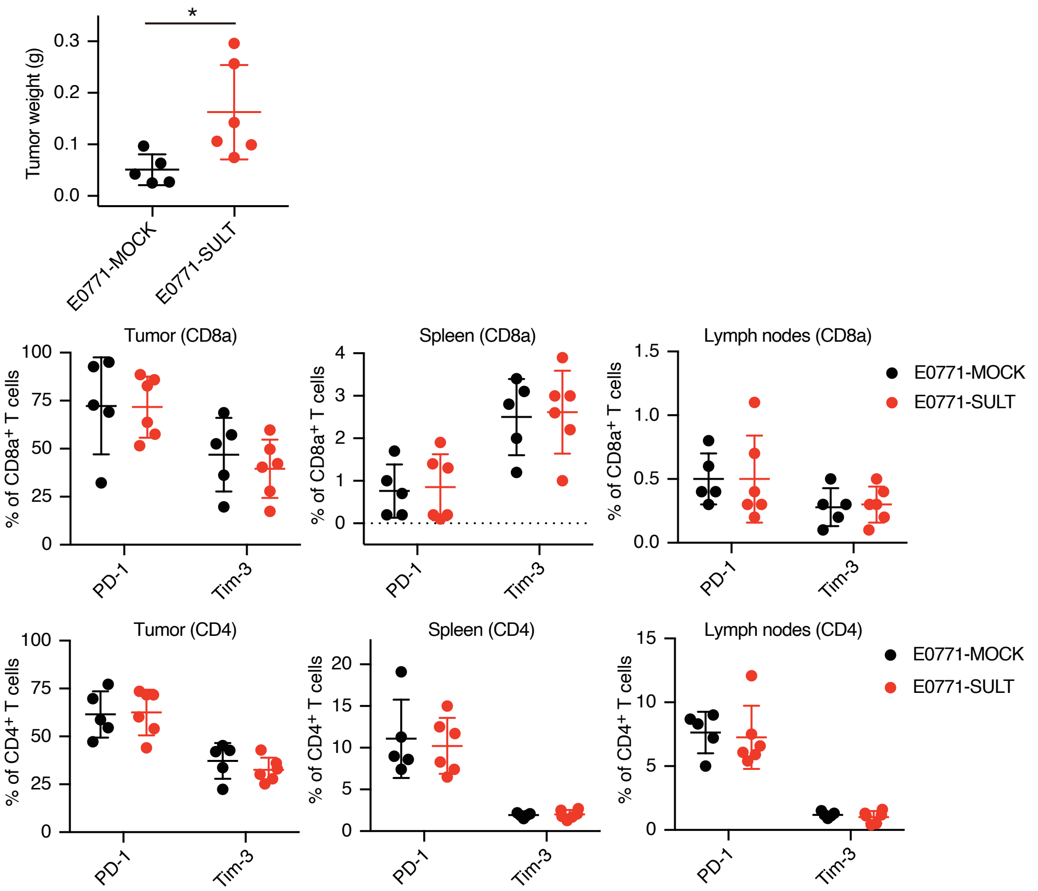
*
Tumor weight (g)
0.0
0.1
0.2
0.3
E0771-MOCK
E0771-SULT
Tumor (CD8a)
Spleen (CD8a)
Lymph nodes (CD8a)
% of CD8a+ T cells
E0771-MOCK
E0771-SULT
PD-1
Tim-3
Tumor (CD4)
Spleen (CD4)
Lymph nodes (CD4)
% of CD4+ T cells

Fig. S4

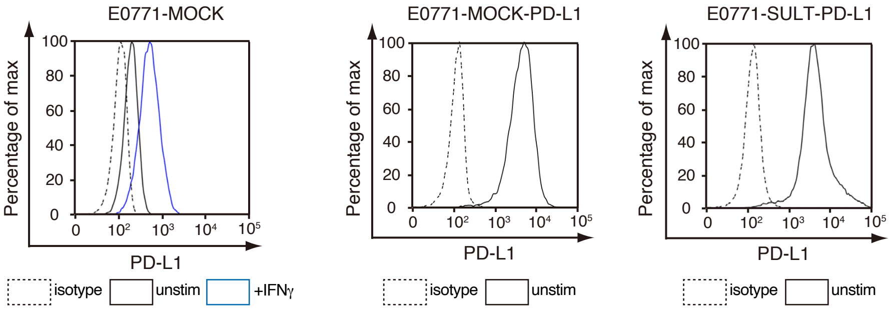
E0771-MOCK
E0771-MOCK-PD-L1
E0771-SULT-PD-L1
Percentage of max
0
20
40
60
80
100
0
10^2
10^3
10^4
10^5
PD-L1
isotype
unstim
+IFNγ

Fig. S5

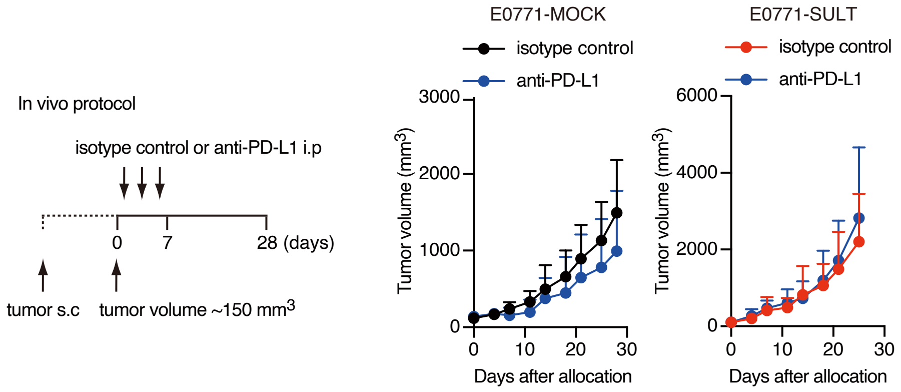
In vivo protocol
isotype control or anti-PD-L1 i.p
0
7
28 (days)
tumor s.c
tumor volume ~150 mm3
E0771-MOCK
isotype control
anti-PD-L1
Tumor volume (mm3)
Days after allocation
E0771-SULT
isotype control
anti-PD-L1
Tumor volume (mm3)
Days after allocation

Fig. S6

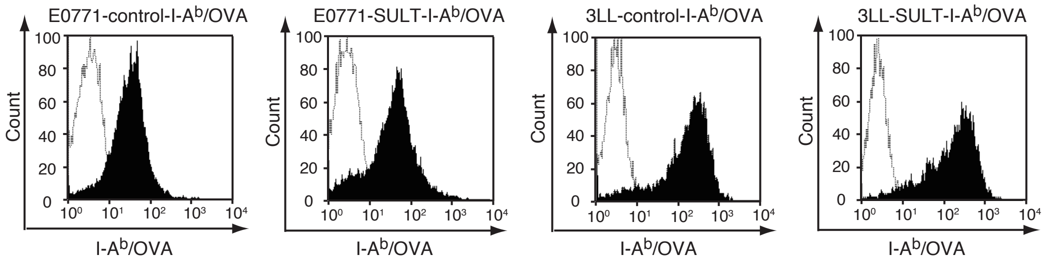
E0771-control-I-Ab/OVA
E0771-SULT-I-Ab/OVA
3LL-control-I-Ab/OVA
3LL-SULT-I-Ab/OVA
Count
100
80
60
40
20
0
10⁰ 10¹ 10² 10³ 10⁴
I-Ab/OVA

Fig. S7

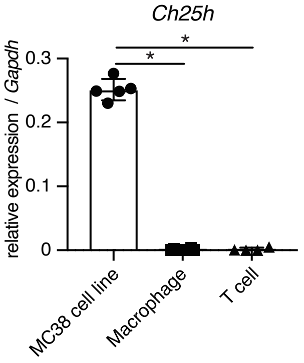
Ch25h
relative expression / Gapdh
0.3
0.2
0.1
0
*
*
MC38 cell line
Macrophage
T cell

Fig. S8

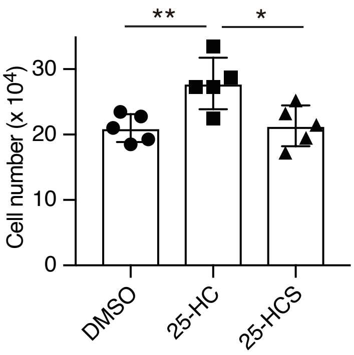

Fig. S9

Table S1. Mass cytometry antibodies panel design

Metal	Mass	Antibody	Clone	Vendor
Y	89	CD45	30-F11	Fluidigm
Pr	141	Ly-6G	1A8	Fluidigm
Nd	142	CD11c	N418	Fluidigm
Nd	144	CD45R (B220)	RA3-6B2	Fluidigm
Nd	145	CD4	RM4-5	Fluidigm
Nd	146	F4/80	BM8	Fluidigm
Nd	148	CD11b (Mac-1)	M1/70	Fluidigm
Sm	149	CD19	6D5	Fluidigm
Nd	150	Ly-6C	HK1.4	Fluidigm
Eu	151	CD25 (IL-2R)	3C7	Fluidigm
Sm	152	CD3ε	145-2C11	Fluidigm
Eu	153	CD274 (PD-L1)	10F.9G2	Fluidigm
Sm	154	CD152 (CTLA-4)	UC10-4B9	Fluidigm
Gd	158	IL-10	JES5-16E3	Fluidigm
Tb	159	CD279 (PD-1)	RMP1-30	Fluidigm
Dy	162	TNFα	MP6-XT22	Fluidigm
Dy	164	LAP (TGFβ)	TW7-16B4	Fluidigm
Ho	165	IFNγ	XMG1.2	Fluidigm
Er	167	IL-6	MP5-20F3	Fluidigm
Er	168	CD8a	53-6.7	Fluidigm
Tm	169	CD206 (MMR)	C068C2	Fluidigm
Er	170	CD161 (NK1.1)	PK136	Fluidigm
Yb	171	CD80 (B7-1)	16-10A1	Fluidigm
Yb	172	Perforin	OMAK-D	Fluidigm
Yb	173	Granzyme B	GB11	Fluidigm
Yb	174	CD223 (LAG-3)	C9B7W	Fluidigm
Lu	175	CD38	90	Fluidigm
Yb	176	CD278 (ICOS)	7E.17G9	Fluidigm
Bi	209	I-A/I-E	M5/114.15.2	Fluidigm
